# Near-field sub-diffraction photolithography with an elastomeric photomask

**DOI:** 10.1038/s41467-020-14439-1

**Published:** 2020-02-10

**Authors:** Sangyoon Paik, Gwangmook Kim, Sehwan Chang, Sooun Lee, Dana Jin, Kwang-Yong Jeong, I Sak Lee, Jekwan Lee, Hongjae Moon, Jaejun Lee, Kiseok Chang, Su Seok Choi, Jeongmin Moon, Soonshin Jung, Shinill Kang, Wooyoung Lee, Heon-Jin Choi, Hyunyong Choi, Hyun Jae Kim, Jae-Hyun Lee, Jinwoo Cheon, Miso Kim, Jaemin Myoung, Hong-Gyu Park, Wooyoung Shim

**Affiliations:** 1 Department of Materials Science and Engineering, Yonsei University, Seoul, 120-749 Republic of Korea; 2 Center for Multi-Dimensional Materials, Yonsei University, Seoul, 03722 Republic of Korea; 3 LCD TV Panel design team 2, LG Display Co., Ltd., Gyeonggi-do, 413-811 Republic of Korea; 4 Center for NanoMedicine, Institute for Basic Science (IBS), Seoul, 03722 Republic of Korea; 5 Yonsei-IBS Institute, Yonsei University, Seoul, 03722 Republic of Korea; 6 Department of Physics, Korea University, Seoul, 02841 Republic of Korea; 7 Center for Safety Measurement, Korea Research Institute of Standards and Science (KRISS), Daejeon, 34113 Republic of Korea; 8 School of Electrical and Electronic Engineering, Yonsei University, 50 Yonsei-ro, Seodaemun-gu, Seoul 03722 Republic of Korea; 9 Department of Physics and Astronomy, and Institute of Applied Physics, Seoul National University, Seoul, Republic of Korea; 10 R&D Center, LG Display, LG Science Park, Seoul, 07796 Korea; 11 Department of Electrical Engineering, Pohang University of Science and Technology (POSTECH), Pohang, 37673 Republic of Korea; 12 School of Mechanical Engineering, Yonsei University, Seoul, 03722 Republic of Korea; 13 Department of Chemistry, Yonsei University, Seoul, 03722 Republic of Korea

**Keywords:** Design, synthesis and processing, Surface patterning, Lithography

## Abstract

Photolithography is the prevalent microfabrication technology. It needs to meet resolution and yield demands at a cost that makes it economically viable. However, conventional far-field photolithography has reached the diffraction limit, which imposes complex optics and short-wavelength beam source to achieve high resolution at the expense of cost efficiency. Here, we present a cost-effective near-field optical printing approach that uses metal patterns embedded in a flexible elastomer photomask with mechanical robustness. This technique generates sub-diffraction patterns that are smaller than 1/10^th^ of the wavelength of the incoming light. It can be integrated into existing hardware and standard mercury lamp, and used for a variety of surfaces, such as curved, rough and defect surfaces. This method offers a higher resolution than common light-based printing systems, while enabling parallel-writing. We anticipate that it will be widely used in academic and industrial productions.

## Abstract (web summary)

Photolithography is an established microfabrication technique but commonly uses costly shortwavelength light sources to achieve high resolution. Here the authors use metal patterns embedded in a flexible elastomer photomask with mechanical robustness for generation of subdiffraction patterns as a cost effective near-field optical printing approach.

## Introduction

The prevailing technology to fabricate semiconductor devices is photolithography. It entails flood-exposing a wafer, coated with a photosensitive polymer, with ultraviolet light through a mask^1^. Currently, the state-of-art photolithography in the industry adopts projection printing, which requires optical elements to focus the mask image onto the wafer surface to achieve a high resolution. It offers low defect density, high registration, high performance, and sub-10 nm node resolution by using extreme ultraviolet (EUV)^1,2^. However, because of its far-field working principle, the resolution of the projection optics is diffraction-limited. It has also been challenging to design complex optics and short-wavelength light source capable of projecting microscale images onto a large-scale wafer while maintaining both high resolution and cost-effectiveness.

In contact printing, a mask is put in physical contact with a photoresist-coated wafer and thus, the resolution of the feature is enhanced by decreasing the gap between the photomask and photoresist surface^3^. If the gap decreases within near-field regime, the photoresist layer is exposed to evanescently decaying components in the near-field^4^. This evanescent wave contains high spatial frequency components and thus, provides higher resolution of features beyond the wavelength of light source without any focusing elements^5^. As the evanescent wave decays exponentially, the mask should be kept in intimate contact with the photoresist surface within less than several tens of nanometer. Hence, flexible photomasks, such as silicon nitride membranes^6^ and thin fused silica^7^, have been proposed to allow conformal contact with the substrate. However, these approaches are vulnerable to local strain which could be caused by dust particles and discontinuous regions on the substrate. Without brittle materials, elastomeric masks utilize a phase shift originated from the structure of elastomer and provide high resolution and mechanical robustness^8,9^. However, they lack the capability to pattern arbitrary shapes, which limits them from being a versatile lithographic tool. To this end, sub-diffraction photolithography using scanning probe-based optical printing^10,11^, high-throughput scanning probe nanolithography^12,13^, and the understanding of photoresist chemistry^14–16^ have been developed for writing arbitrary patterns composed of diffraction-unlimited features. A photolithography technique that provides mechanical robustness, sub-diffraction resolution of arbitrary-shaped features, and compatibility for a variety of surfaces at low cost would be considered ideal.

Here, we report a photolithography technique that achieves these goals by using an elastomeric photomask with a mechanically stable metal absorber that allows reliable conformal contact and thus, near-field alignment with the surface. We analyze the mechanical behavior of metal patterns on a soft substrate and derive a design rule to prevent the mechanical vulnerability of metal patterns against local strain on the mask. In comparison with other image-transfer techniques, our technique provides potential advantages in terms of mechanical robustness, sub-diffraction scaling, and applicable surfaces at low cost. Our technique prints features below the diffraction limit over a large area on curved, rough, and defect surfaces without causing a mechanical damage on mask or compromising the depth of focus. The combination of near-field capability for sub-diffraction pattern generation with elastomeric characteristic of the mask provides a simple, flexible, and low-cost lithographic tool that has the potential to replace EUV lithography.

## Results

### Designing a soft photomask for reliable sub-diffraction photolithography

The fundamental design principle of the soft photomask is to provide a conformal contact on photoresist surface while containing a binary amplitude pattern that is similar to conventional chromium-glass photomask. These requirements could be met by embedding a metal absorber in the soft substrate. Several studies^17,18^ have been proposed to compose a metal pattern on polydimethylsiloxane (PDMS) substrate, which has excellent flexibility^19^ and high transmittance in the wavelength >300 nm^20^. Although their resolutions are not in sub-wavelength scale, these approaches demonstrate successful photolithographic performance. However, the mechanical stability of metal pattern is hardly secured. Soft photomask usually suffers from local strain owing to low Young’s modulus of substrate and leads to failure in brittle metal pattern. When the mask is repeatedly used, this mechanical vulnerability results in deformation in pattern and short lifetime of the photomask and loses its merits of photolithography with respect to reliability and cost-efficiency. The key to realizing practical contact printing is to guarantee the stability of metal patterns on soft photomask.

**Fig. 1:**
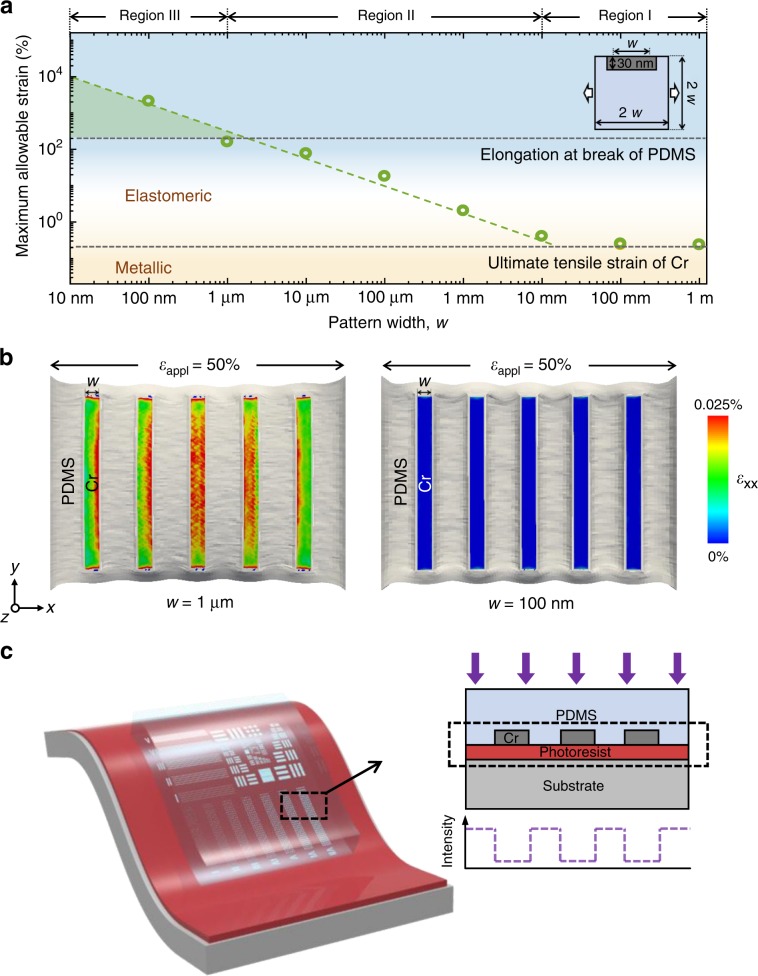
Concept of optical contact lithography technique using a metal-embedded elastomeric photomask. **a** Numerical simulation results (dots) of the maximum allowable strain of a metal pattern embedded in an elastomeric substrate at different metal pattern widths (*w*). The dashed line shows a fitted line of simulation results. The inset shows schematic illustration of model for simulation model. **b** Simulated results of strain (*ε*_xx_) distribution in a Cr line array pattern embedded in a PDMS substrate for *w* = 1 µm (left) and *w* = 100 nm (right). **c** Schematic illustration of contact printing with a soft photomask (left) and light intensity profile (right).

If we consider metal-embedded soft substrate as a composite material composed of soft matrix and a sheet-like hard additive, the mechanical behavior of metal pattern is affected by the surrounding soft matrix and geometry of the metal pattern itself^21^. We performed numerical simulations of the maximum allowable strain for metal patterns embedded in an elastomeric mask plate with different width of metal pattern (Fig. 1a). The Young’s modulus ratio in this study was that of chromium (Cr) and PDMS (*E*_*PDMS*_/*E*_*Cr*_ ~ 10^−5^). The maximum allowable strain was defined as a measure of the strain that caused plastic deformation of the metal patterns embedded into the elastomeric mask plate^22^. In this simulation, we were able to distinguish between three separate regions: (i) region I with a pattern width (*w*) > 10 mm with identical separations of *w* (inset of Fig. 1a), where the mask plate followed metallic strain behavior, (ii) region II with *w* between 10 mm and 1 μm, where the mask plate followed elastomeric strain behavior, and (iii) region III with *w* below 1 μm, where the mask plate endured more than upper limit strain of elastomer. This indicates that by decreasing *w*, the maximum allowable strain of a metal pattern gradually increased. As a result, the mask plate can bear the strain even above the fracture stain of the elastomer. At sub-micrometer length, the strain applied on the metal pattern was dramatically alleviated and could withstand 200% strain. This exceeds an elongation at the break of PDMS, which compares well with previous studies^21,23^. This mechanical behavior is particularly useful at sub-diffraction scale because it results in less mechanical damage when the elastomeric photomask gets in contact with the surface.

To confirm this mechanical design rule that we found based on numerical simulations, we also performed analytical calculations of a strain-isolation behavior of metal patterns embedded in an elastomeric substrate. For this, it was reasonable to utilize the shear-lag model^24^ where the strain applied to the elastomeric matrix was transferred to a metal pattern through shear stress at the interface. According to this model, we found that the ratio of the maximum tensile strain for the metal thin film to the strain applied to elastomeric substrate is

1 $$\frac{{\varepsilon _{{\mathrm{max}}}}}{{\varepsilon _{{\mathrm{appl}}}}}{\mathrm{ = 1}} - \frac{1}{{{\mathrm{cosh}}\left( {\sqrt {\frac{{G_sw^2}}{{4E_{\mathrm{{m}}}{Tt}}}} } \right)}}$$

where *G*_s_ is the shear modulus of a substrate, *E*_m_ the Young’s modulus of the metal thin film, *w* the width of metal thin film, *t* the thickness of the metal thin film, *T* the effective thickness of the substrate (see Supplementary Text). Equation 1 shows that the strain transferred to the metal thin film is determined by the geometric parameters (i.e., width and height) of the metal film in addition to the stiffness of film and substrate. This means that when the thickness of the metal film remains constant, the maximum allowable level of strain in the metal thin film increases with decreasing width of the metal thin film, leading to similar characteristics as simulated in Fig. 1a. Furthermore, we observed this tendency using 3D finite element method (FEM) simulation of a Cr line array pattern embedded in a PDMS substrate (Fig. 1b and Supplementary Fig. 1). The strain transferred to a Cr line array reduced as the width of a Cr line decreased from 1 μm to 100 nm, which is also in good agreement with the shear-lag model^24^.

In order to perform photolithography at near-field illumination, we fabricated a photomask with metal pattern arrays embedded in a transparent spring-like elastomer plate. This key innovation allowed for the entire photomask surface to be in direct contact with a photoresist-coated wafer surface (Fig. 1c). The soft photomask architecture was comprised of an opaque array of a Cr pattern, which was embedded in an elastomeric mask plate. The elastomeric backing significantly reduced the mechanical damage when the mask was pressed into tight contact with the photoresist-coated surface. This reduced debris and undesired voids enabling full contact between mask and surface, and thus, improving the resolution across the wafer.

To create the Cr pattern into PDMS as a mask, we used standard electron-beam lithography, deposition and lift-off processes. Briefly, a 200 nm Ni layer deposited by sputtering on the Si/SiO_2_ served as a sacrificial layer (Supplementary Fig. 2). After 30 nm-thick Cr layer was patterned by electron-beam lithography on the Si/SiO_2_/Ni surface. We casted a PDMS layer on this surface followed by a curing process. Following etching of the Ni film, the Cr pattern-embedded photomask with elastomeric backing was well separated from the Si/SiO_2_ surface. It is worth noting that a failure of Cr transfer from the Ni film to PDMS and Cr debonding from PDMS can occur because of the weak adhesion between Cr and PDMS (Supplementary Fig. 3), particularly when the Cr pattern is small^22^. To avoid this, the Cr pattern was exposed to O_2_ plasma before coating the PDMS layer, and enough time was spent to thoroughly etch the sacrificial Ni layer.

### Characterization of the Cr patterns in the soft photomask

**Fig. 2:**
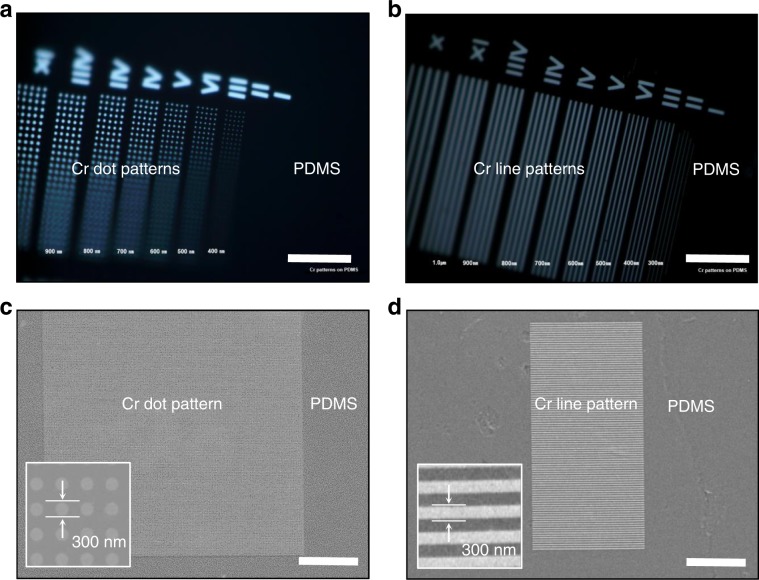
Cr patterns in the soft photomask are highly accurate and crack-free. **a**, **b** Optical microscopy images of soft photomask containing Cr dot (**a**) and line (**b**) patterns. Scale bar: 20 μm. **c**, **d** Scanning electron microscopy (SEM) images of Cr dot (**c**) and line (**d**) patterns. The insets show magnified images. Scale bar: 10 μm.

In order to ensure that the Cr patterns embedded in PDMS were intact after the mask fabrication procedure, we used optical microscopy to obtain images of the soft photomask with Cr dot (Fig. 2a) and line patterns (Fig. 2b). Because PDMS is transparent, one can visually align the chromium patterns with respect to the wafer surface, which makes lithography protocol straightforward to use. We observed no cracking for both in PDMS embedded Cr patterns. Usually, when deposited directly on the PDMS surface, cracking is easily induced due to the thermal expansion coefficient mismatch^25,26^. We also observed that the soft photomasks were not rigid, placing the part of Cr patterns of these optical images out of focus (Figs. 2a, b).

To assess the Cr patterns of the soft photomasks, we conducted scanning electron microscopy (SEM) analysis of the soft photomasks (Figs. 2c, d). We found that our fabrication protocol yielded an array of uniform and continuous Cr patterns embedded in PDMS. The dot pattern with 300-nm dots separated edge-to-edge by 300 nm were well defined into PDMS surface and remarkably uniform with a pitch distance of 600 nm (Fig. 2c). We also achieved a line pattern yield of >99% without cracks using our fabrication procedure (Fig. 2d). The average pitch was 600 nm, demonstrating that our soft photomask does not exhibit any significant defects, such as cracks. This agrees with our numerical simulation results as shown in Fig. 1a. In principle, the dot and line pitch distance in the PDMS mask plate could be reduced to less than 10 nm using electron-beam lithography. To evaluate the potential for sub-50 nm optical photolithography, however, a pitch distance of 100 nm on the mask is sufficient.

### Image transfer properties and feature size control using the soft photomask

**Fig. 3:**
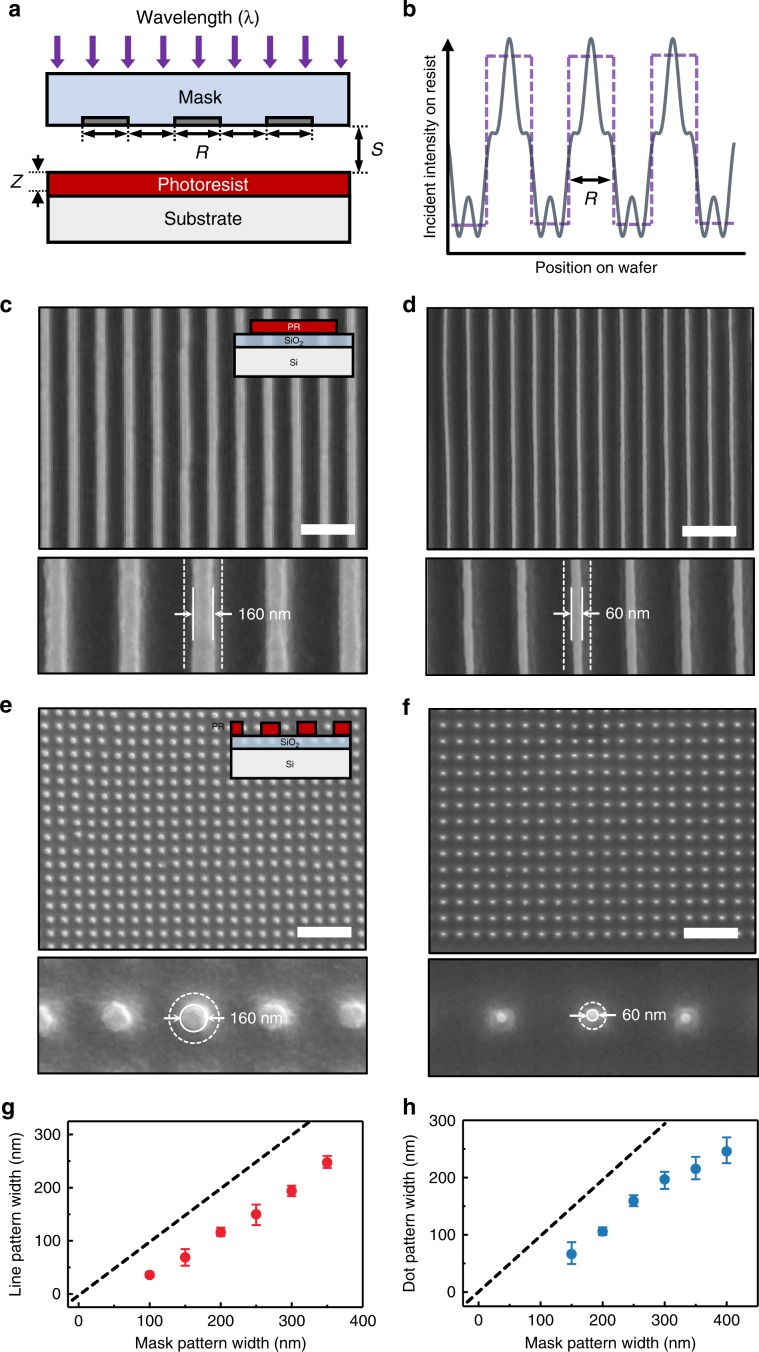
Cr pattern in soft mask can be transferred to photoresist features with great size control. **a** Schematic illustration of shadow printing mode optical lithography. *R*, *S,* and *Z* are the width of pattern, the gap between mask and photoresist surface and the thickness of photoresist, respectively. **b** Light-intensity distribution at the surface patterned by contact printing with gap (*S* > 0, solid gray line) and without gap (*S* = 0, dotted purple line). **c**, **d** SEM images of photoresist line features defined by Cr patterns of 300 nm (**c**) and 150 nm (**d**) in width. Dotted lines indicate the size of the chrome pattern in soft mask. Scale bar: 1 μm. **e**, **f** SEM image of photoresist dot features defined by Cr patterns of 300 nm (**e**) and 150 nm (**f**) in diameter. Dotted circles indicate the size of the chrome pattern in soft mask. Scale bar: 2 μm. **g** Photoresist line feature variation (red dots) depending on Cr pattern width. **h** Photoresist dot feature variation (blue dots) depending on Cr pattern diameter. In (**g**, **h**), error bars indicate a maximum and minimum value in the experiment results and the dotted line is guiding line (*y* *=* *x*).

In order to investigate the image transfer properties of the soft photomask, it was brought into contact with a silicon substrate that was pre-coated with a positive photoresist (Dow Electronic Materials, MEGAPOSIT SPR510A) and exposed to UV light. PDMS, a substrate of our soft mask, has high transmittance and UV stability in the wavelength >300 nm (Supplementary Fig. 4). Incident UV light through soft photomask can be transferred onto the photoresist layer without loss of light intensity. Ideal image transfer would produce a pattern of light at the wafer surface. However, the actual intensity pattern deviated considerably from this square-wave pattern (Fig. 3a, b). This deviation is owing to diffraction, which can be minimized by hard contact printing. In this context, we investigated the feature size control by varying the mask pattern pitch using the same image transfer experiment. First, we characterized how decreasing the Cr pattern size in the mask affected the resulting feature size. For this, we coated photoresist with a 1 μm-thick photoresist layer and exposed them to 350–450 nm UV light from a mercury-vapor light source, followed by development process (Developer, Tetramethylammonium hydroxide 2.38 wt%, nepes, CPD-BD). Soft photomask was attached to a dummy glass and mounted onto a conventional mask aligner (MIDAS SYSTEM, MDA-400S) to control the location of photomask and expose UV light (Supplementary Fig. 5). After development, we observed photoresist line features of a width of 160 nm (Fig. 3c) and 60 nm (Fig. 3d) when Cr line patterns of 300 nm and 150 nm in width had been used in the soft photomask, respectively. These features, particularly for 60-nm features, could be transferred although they are much smaller than the wavelength of the light source. This can be attributed to gapless contact between the mask and the surface, where hard contact printing allows near ideal image transfer (*S* = 0, and see Figs. 3a, b). Similarly, using Cr dot patterns of 300 and 150 nm in diameter, we observed dot features of an average diameter of 160 nm (Fig. 3e) and 60 nm (Fig. 3f), respectively. The developed photoresist line width was 45% smaller than the Cr line width in the soft photomask (Fig. 3g and dots for Fig. 3h), primarily because of the diffraction in photoresist layer and partial photoresist loss during development^27,28^. The novolac-based photoresist used here, is slightly soluble in alkaline developer even when it is not exposed to UV light^29^. Combined with the diffraction inside photoresist, it caused the overdevelopment of small features. This overdeveloping phenomenon can be managed with exposure dose–development time process window to reproduce the shrunk features^30^. In our study, at the given parameters of a resist thickness of 1 μm, an exposure energy of 17 mW/cm^2^, an exposure time of 5 s, and a developing time of 2 s, the contrast between developed/undeveloped regions was maintained, while the thickness of remaining photoresist layer decreased to 300 nm.

**Fig. 4:**
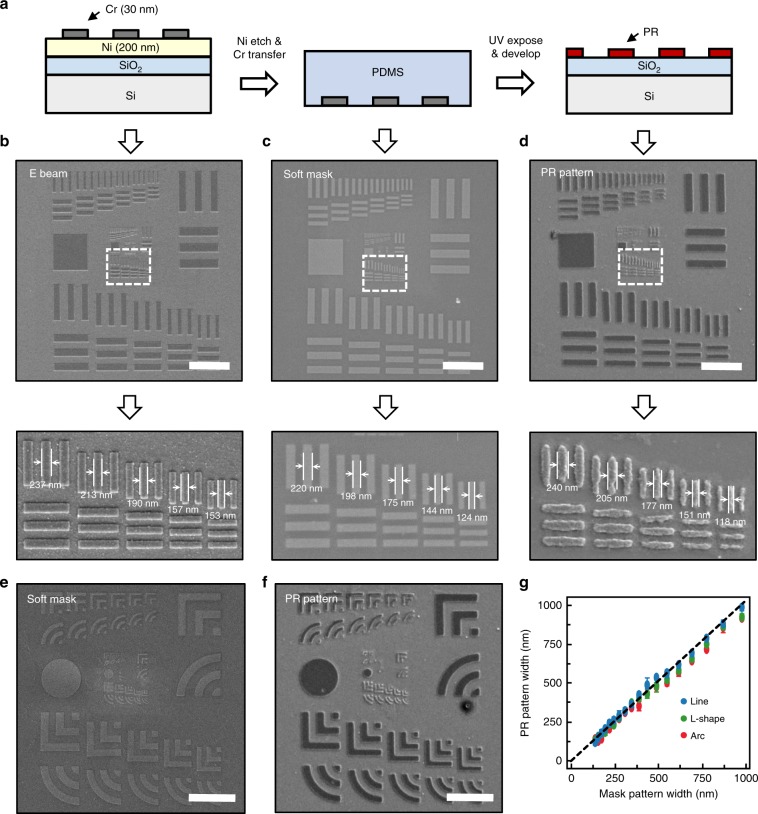
Soft photomask to quantitatively resolve sub-diffraction surface features. **a** Schematic illustration of transferring steps during optical lithography by the soft photomask. **b**–**d** SEM images of the 1951 USAF resolution test chart at each transferring step illustrated in (**a**), i*.*e., defining Cr patterns by e-beam lithography (**b**), Cr patterns embedded in a PDMS mask (**c**) and the photoresist pattern (**d**). The lower panels show magnified images of the parts of the upper panel images indicated with dotted squares. Scale bar: 5 μm. **e** SEM image of modified 1951 USAF resolution test chart for arc and L-shaped pattern in soft photomask. Scale bar: 5 μm. **f** SEM image of patterned photoresist feature corresponding to (**e**). Scale bar: 5 μm. **g** Average width of patterned photoresist features depending on Cr pattern width. Error bars indicate a maximum and minimum value in the experiment results and the dotted line is guiding line (*y* *=* *x*).

To determine the quantitative feature resolution using our soft photomask, we used a 1951 USAF (U.S. Air Force) resolution test chart as the mask pattern (Fig. 4). The full standard pattern consisted of six groups consisting of six elements each, and thus, 36 target elements in total (Supplementary Fig. 6 and Supplementary Table 1). In this experiment, we incorporated a 30 nm-thick photoresist layer to minimize far-field diffraction through the resist and thereby investigated a near-field lithography capability of our technique. A thin photoresist layer was prepared by diluting a photoresist (MicroChem, S1805) with propylene glycol monomethyl ether acetate (DAEJUNG) in 1:9 volume ratio. In a typical experiment, electron-beam lithography was performed to define Cr patterns according to the USAF resolution test chart. The patterns were transferred into PDMS and then onto a photoresist (Fig. 4a). In this evaluation test, the electron-beam pattern (Fig. 4b) resulted in an accurate photomask (Fig. 4c) and photoresist pattern (Fig. 4d). In order to resolve individual features, the images were magnified in the portion of the USAF test chart that corresponds to the elements 2, 3, 4, 5, 6 and group number 11. In all the images, individual line features can be clearly distinguished, allowing us to determine the smallest feature width of ~118 nm in the photoresist pattern (Fig. 4d). We repeated an evaluation test for arc and L-shaped features and observed similar results (Fig. 4e, f, and Supplementary Fig. 7). All the photoresist patterns showed excellent correspondence with soft photomask, demonstrating high accuracy and precision of near-field exposure through our technique (Fig. 4g). We observed the advantage of softmasks in comparison with the results of glass masks (Supplementary Figs. 8a, b). We patterned same resolution test charts with a 30 nm-thick chromium glass photomask. Contrary to softmask, photoresist features exhibited a blurred shape (Supplementary Figs. 8c, d). We determined the minimum distinguishable line width of 1140 nm at hard contact pressure 53.2 kPa, which is about 10 times larger than that of soft mask. This implies that the glass photomask could not be in full contact with the photoresist surface while causing considerable far-field diffraction between the glass mask and photoresist layer. The resolution capability of shadow printing is given by

2 $$2R \,{\mathrm{ = 3}}\left[ {\lambda \left( {s + \frac{1}{2}z} \right)} \right]^{\frac{1}{2}}$$

where 2*R* is the grating period, *λ* is the wavelength of the exposing radiation, *s* is the gap distance between the mask and the photoresist surface, and *z* is the photoresist thickness^3^ (Fig. 3a). According to Eq. 2, the expected gap between glass mask and photoresist surface is 907 nm when we assume monochromatic radiation at 365 nm. We inferred that the gap between wafer and glass photomask resulted from non-planarity of substrate^31^, which hinders the nanoscale patterning unless we use much shorter wavelength light source than target resolution. The above results confirm that soft photomask made a conformable and gapless contact with photoresist-coated substrate and thus, exhibited the distinctive advantage in patterning resolution.

### Patterning on curved, rough, and defect surfaces

**Fig. 5:**
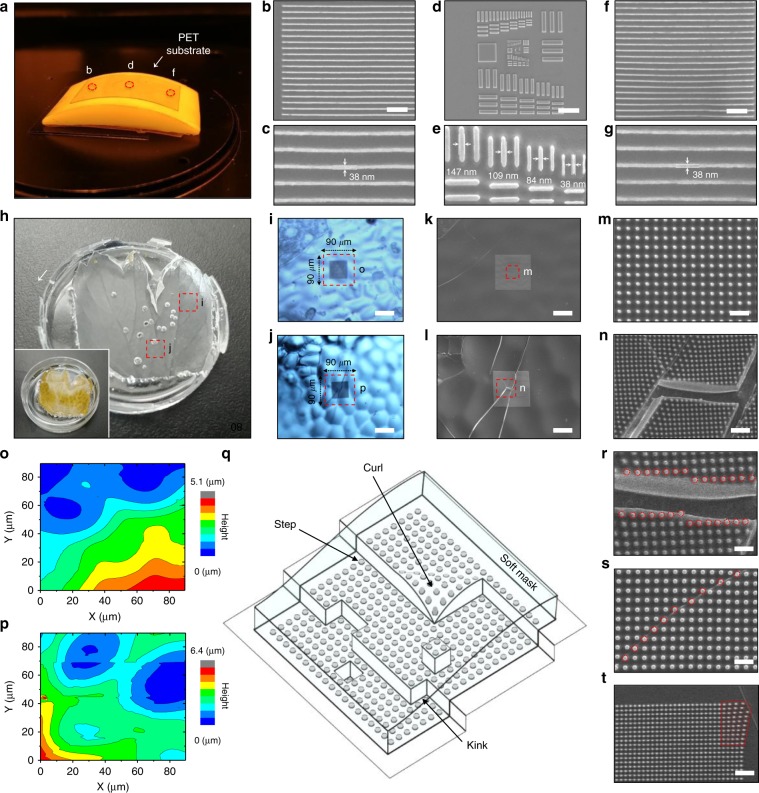
Soft photomask resolves curved, rough and defect surfaces. **a** Photograph of PET substrate on a curvilinear Teflon surface. **b**–**g** SEM images of the photoresist patterns at the three points (**b**, **d**, **f**) indicated in (**a**). The upper panel images show images with lower magnification, and the lower panel images with higher magnification. Scale bars: 1 μm for (**b**, **f**), 5 μm for (**d**). **h** Photograph of leaf-shaped PDMS as a substrate. The inset shows a photograph of a real leaf that was used as molding template. **i, j** Optical microscopy images of photoresist features on the parts of the substrate indicated with red dotted rectangles in exhibiting continuous (**i**) and discrete (**j**) surfaces. Scale bars: 50 μm. **k**–**n** SEM images of photoresist dot pattern on the continuous (**k**) and discrete (**l**) surfaces. **m, n** Show magnified images of (**k**) and (**l**), respectively, of the part of the surface as indicated by red dotted rectangles. Scale bars: 25 μm for (**k**, **l**), 1 μm for (**m**), 2 μm for (**n**). **o**, **p** AFM images of the continuous (**o**) and discrete (**p**) parts of the surfaces as indicated by red dotted rectangles in (**i**, **j**). **q** Schematic illustration of a soft substrate on a surface with step, curl and kink defects. **r**–**t** SEM images of photoresist dot feature at kink (**r**), step (**s**) and curl (**t**) on a surface. Scale bars: 1 μm for (**r**, **s**), 2 μm for (**t**).

To test the capability of our soft photomask in creating structures on the surface with curvature, we used our soft photomask to create photoresist patterns on a Teflon surface with a radius of curvature of 250 mm (Fig. 5a). In this experiment, we spin-coated photoresist with 600-nm thickness in order to form stable photoresist layer on curved surface. Under a 400 nm UV exposure for 5 s, soft photomask was placed onto the photoresist-coated Teflon to fabricate photoresist line patterns (indicated by b and f) and the USAF resolution test chart (indicated by d). We found that all patterns were extremely homogenous (Fig. 5b‒g). The highest line resolution was <40 nm (Fig. 5c, e, g) using the Cr line width of 100 nm, approaching to 1/10 of the wavelength of the diffraction limit. Although the thinnest line width is partially due to overdevelopment of thick photoresist, it is reproducible with the same exposure and development conditions. We note that when Teflon is used as the surface, the reflected light from the surface is minimized^32–34^, compared to a silicon surface; as a result, this may lead to more homogeneous and clearly defined resulting photoresist line patterns. In contrast to conventional photolithography with a rigid photomask, our technique can provide the capability to pattern non-flat surfaces. This advantage bypasses the need for (and the costs associated with) projection optics including lens elements to focus the mask image onto the wafer surface needed in order to overcome the diffraction limit.

We then investigated the suitability of our soft photomask for surfaces with a higher degree of roughness, in particular with height differences in the microscale, which conventional photolithography cannot resolve. For this purpose, we prepared a rough surface using a leaf (inset of Fig. 5h) as a mold, and then peeled PDMS off the mold after a curing process (Fig. 5h). Two types of rough surfaces were prepared: A topographically continuous one (Fig. 5i) and a discrete one with surface defects (Fig. 5j). The roughness of these surfaces was determined from 90 × 90 μm^2^ AFM images. The maximum peak-to-valley heights of the continuous and discrete surfaces were measured to 6 μm (r.m.s roughness ~1.2 μm, Fig. 5o) and 10 μm (r.ms. roughness ~1.1 μm, Fig. 5p), respectively. Under 400-nm UV exposure for 5 s and development, the continuous surface showed clear 50 × 50 μm^2^ features consisting of 10,000 dots (100 × 100 dot array, Fig. 5k). Each dot was 200 ± 7 nm in diameter with a pitch distance of 500 nm (Fig. 5m).

Interestingly, on the discrete surface with surface defects (Fig. 5l), the 100 × 100 dot pattern was also clearly defined, without any apparent dots missing (Fig. 5n). From a materials science perspective, the discrete surface contains typical surface defects such as steps, kinks, and curls, as represented schematically in Fig. 5q. We found that no dots in kink (Fig. 5r), step (Fig. 5s), and curl (Fig. 5t) defects were noticeably missing. The ability to transfer the image onto a surface that is not only rough but also comprising surface defects, while exhibiting the sub-diffraction-limited resolution, is a clear advantage of our soft photomask compared to previous methods using rigid masks. We anticipate that our soft photomask approach can enable surface patterning with sub-diffraction-limit resolution and provide a framework for realizing successful miniaturization.

### Multiple feature patterning capability of the soft photomask

**Fig. 6:**
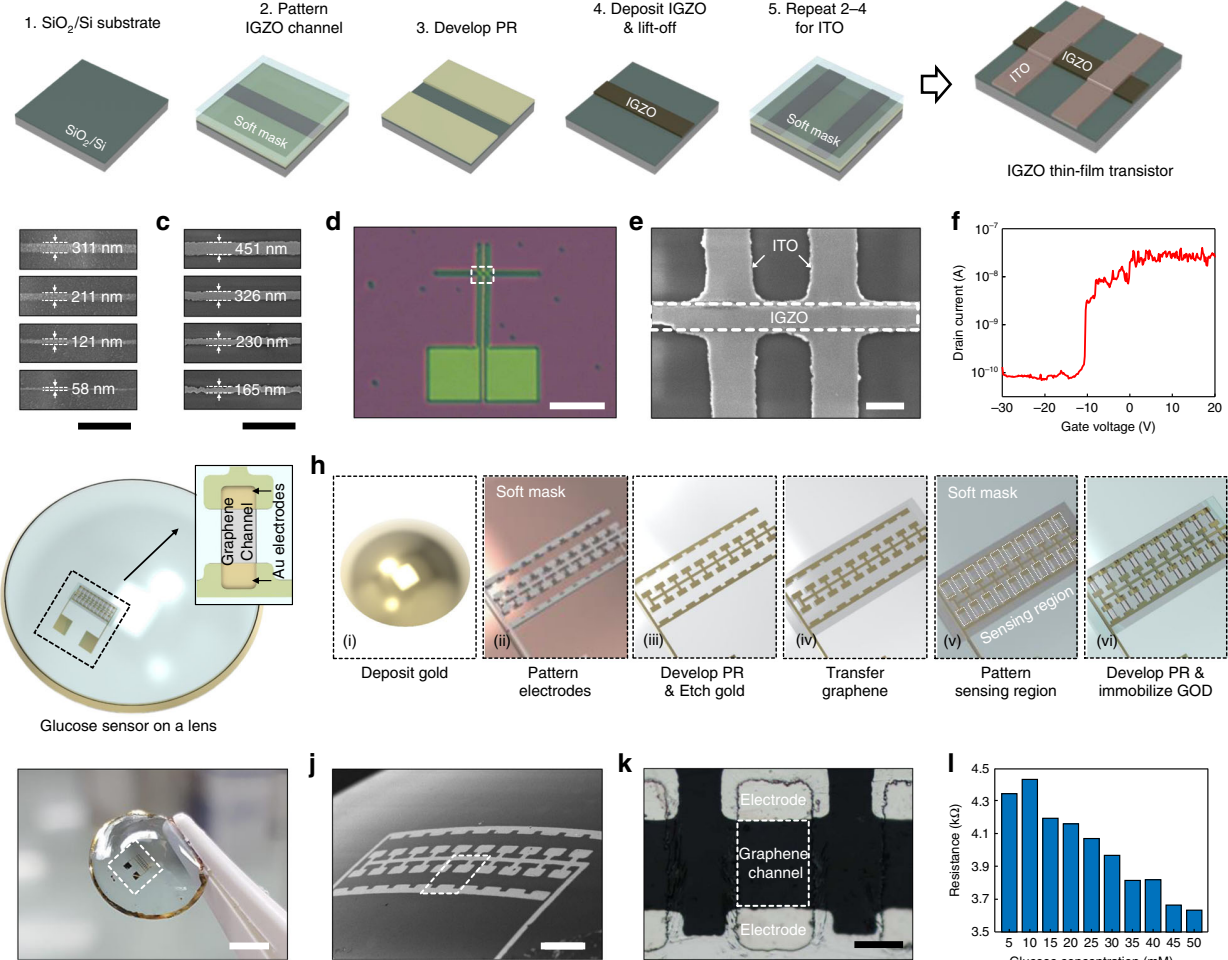
Soft photomask provides generic and multiple feature patterning. **a** Experimental procedure to fabricate an IGZO thin-film transistor. **b**, **c** SEM images of Cr pattern embedded in soft photomask (**b**) and corresponding IGZO pattern (**c**). Scale bars: 2 μm. **d** Optical microscopy image of IGZO thin-film transistor. Scale bar: 10 μm. **e** SEM images of an IGZO channel and ITO electrodes. Scale bar: 500 nm. **f** Transfer curve of an IGZO thin-film transistor. Drain bias is 10.1 V. **g** Schematic illustration of glucose sensor on a contact lens. **h** Fabrication process for glucose sensor directly patterned on a contact lens. **i** Photograph of electrode-patterned contact lens. Scale bar: 3 mm. **j** SEM image of Au electrodes patterned on the contact lens. Scale bar: 200 μm. **k** Optical microscopy image of confined graphene channel and Au electrodes. Scale bar: 50 μm. **l** Electrical resistance of glucose sensor depending on glucose concentration.

We explored multiple patterning capability of soft photomask with several representative applications to verify its relevance as a generic pattern transfer tool. First, we fabricated an indium gallium zinc oxide (IGZO) thin-film transistor^35^. Figure 6a depicts the procedure to fabricate an IGZO thin-film transistor. Using our soft photomask, we executed the photolithography process twice to pattern the IGZO channel and ITO electrodes, respectively. We coated a negative photoresist (DONGJIN SEMICHEM, DNR-L300-40, diluted with cyclopentanone in 1:14 volume ratio) on an SiO_2_ (300 nm)/p-doped Si substrate for the IGZO channel. We exposed UV light onto the substrate using our soft photomask. After the development, we deposited a 20 nm-thick IGZO film followed by lift-off. We repeated the same procedure to pattern 45 nm-thick ITO electrodes. Fig. 6b, c show chrome patterns and corresponding patterned IGZO channels with various line widths. We observed that a minimum line width of the IGZO channel was 165 nm, while the width of patterned IGZO is about 100 nm larger than that of the chrome pattern. This discrepancy could be due to diffraction inside the photoresist layer resulting from its relatively higher thickness. As we coated a 100 nm-thick photoresist layer in this experiment, from Eq. 2, the expected resolution is approximately 200 nm. Although a thick photoresist facilitates easier lift-off, it induces diffraction inside the layer and thus, degrades the resolution of features. This trade-off determines the practical resolution of the process depending on specific conditions of fabrication (e.g., thickness of target material and deposition method). Subsequently, after patterning the IGZO channel and ITO electrodes, we characterized a back-gated IGZO thin-film transistor with a channel width of 240 nm. The transistor was composed of a p-doped silicon substrate as gate (Fig. 6d, e). A transfer curve of the transistor showed typical characteristics of an n-type transistor^36^ while the threshold voltage was negative-shifted at ~10 V (Fig. 6f).

The unique capability of a soft photomask is to directly pattern the features on curved substrates. This is difficult to achieve by conventional projection lithography because of the limitation in depth of focus (DoF). To examine the applicability of our technique to a non-planar device, we fabricated a glucose sensor directly on a commercial contact lens. Recently, a smart contact lens detecting glucose concentrations has been proposed to provide non-invasive diagnosis and real-time monitoring^37–39^. Thus far, photolithography was hardly applied to pattern the device on a contact lens because of its curved shape and imposed transferring process from a planar substrate. The ability of soft photomask to conform to various shapes of a substrate can provide an efficient way to fabricate a sensor on a curved contact lens.

The glucose sensor was composed of Au electrodes and a graphene channel with an immobilized glucose oxidase (GOD) (Fig. 6g). The sensor indicates glucose concentration by resistance change of the graphene channel, which results from oxidation of glucose^39^. Figure 6h depicts a fabrication process of glucose sensor on a contact lens. A 100 nm-thick Au layer with a 10 nm-thick Cr adhesive layer was deposited on a commercial contact lens (NEON, Blue). Cr/Au film was patterned by photolithography followed by etching process to form the electrodes (Fig. 6g, h). We transferred the graphene onto the contact lens and confined the graphene channel by SU-8 photoresist (Fig. 6i). The graphene channel was then treated with 1-pyrenebutanoic acid succinimidyl ester^40^ and functionalized with GOD and catalase. Throughout the fabrication process, we did not observe any distortion on features which could be caused from defocusing of pattern (Fig. 6i–k). Our soft photomask enabled the alignment of metal pattern along the highly curved surface and thus, transferred the features onto the contact lens without loss of focus. In this experiment, we used 1.5 μm-thick metal pattern on the soft mask to secure its durability. Metal patterns with their sizes in hundreds of micrometers experienced severe shear stress from the interface with PDMS substrate when compared to the stress in nanoscale patterns. Thick metal film could compensate for the shear stress resulting from large size of pattern as qualitatively described in Eq. 1 (Supplementary Fig. 9).

After fabrication, we characterized the glucose sensor fabricated on the contact lens. We transferred a drop of glucose solution onto the contact lens and measured the resistance change of the sensor, which depends on concentration of glucose (Fig. 6l). We observed that resistance across graphene channels steadily decreased as the concentration of glucose increased. This concentration-dependent resistance is in agreement with previous studies^38,39^, verifying the working of our fabricated sensors. Glucose sensor on a contact lens is a good example to demonstrate the patterning capability of a soft photomask with various shapes of substrate. It suggests that our technique can provide a simple and versatile tool to fabricate a non-planar device without any additional transferring process.

### Full-color and large-scale printing using soft photomask

**Fig. 7:**
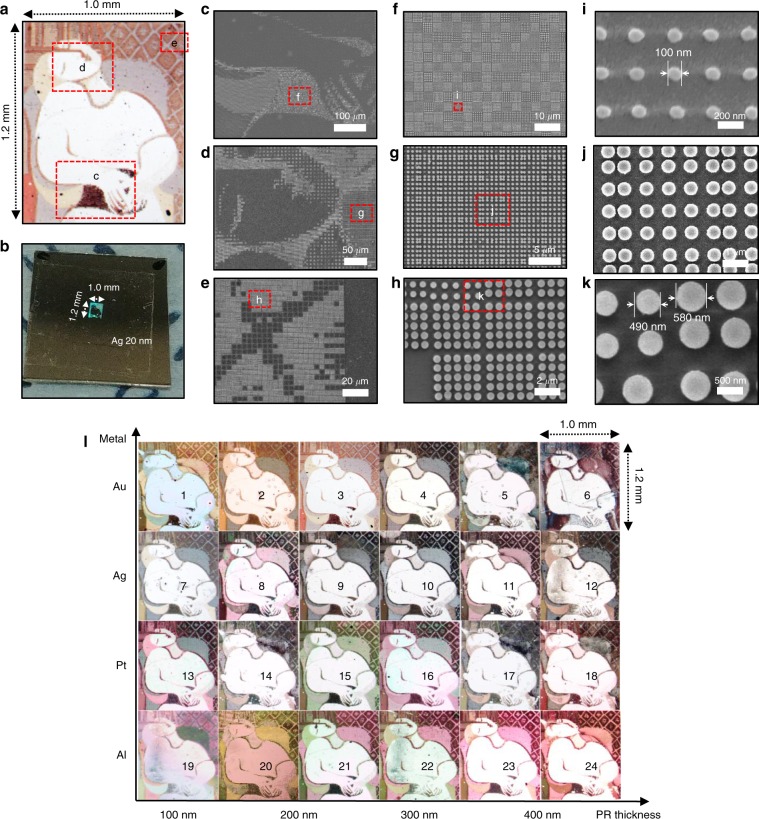
Soft photomask allows for full-color and large-scale printing. **a**, **b** Optical microscope image (**a**) and photograph by mobile phone (**b**) of a photo-realistic Picasso painting produced using plasmonic nanodisks. **c**–**k** SEM images of three different parts of the plasmonic structures indicated in (**a**) at three magnifications, from low (left panels) to high (right panels). Scale bars: 100 μm for (**c**), 50 μm for (**d**), 20 μm for (**e**), 10 μm for (**f**), 5 μm for (**g**), 2 μm for (**h**), 200 nm for (**i**), 1 μm for (**j**), 500 nm for (**k**). **l** Millimeter-scale printing with various colors by a consecutive optical lithography process. The different colors were obtained by changing the thickness of the photoresist in increments of 100 nm and varying the deposited metal species (Au, Pt, Ag, and Al). The original image was converted to plasmonic structures using encoding algorithms. Process and design parameters of each plasmonic image are summarized in Supplementary Table 2.

We evaluated the near-field contact printing capability of our soft photomask—including the ability for reproducible sub-diffraction writing and parallel writing. For this, we used a millimeter scale plasmonic color image with color control. This artificial structural color originates from resonant interactions between visible light and manufactured nanostructures. It is emerging as a solution for ink-free color printing^41–44^, but there still exist difficulties in their large scale implementation because of the size requirement^45–48^. First, we simulated the reflectance of different pixels composed of a regular arrangement of 16–225 nanodisks with various diameters (*d*) and separations (*s*). By changing *d* and *s*, the spectral intensity of each wavelength range in the visible region changed, and each condition was used as encoding data for each pixel (4 μm × 4 μm, Supplementary Fig. 10). To evaluate the capability of our soft photomask to produce a photo-realistic image, we coded color information from bitmap images of a Picasso painting pixel by pixel into position with nanodisks of different *d* and *s* resulting in a 1.2 mm × 1.0 mm image (Fig. 7 and Supplementary Fig. 11). The soft photomask was used to fabricate the photoresist dot pixels (200 nm in height) with desired *d* and *s*, and subsequently, Ag was deposited (20 nm). The color information latent in the gray-scale structures manifested upon deposition of the metal layers (Fig. 7a). It is noteworthy that the resulting image composed of 10,800,000 dots was eventually visible to the naked eye (Fig. 7b). When zooming into different areas of the Picasso image (Fig. 7c‒h) one can distinguish individual dots (Fig. 7i‒k), allowing us to determine the feature size down to 100 nm. To the best of our knowledge, this is the largest plasmonic color structure^49,50^, which demonstrates potential applications of large-area plasmonic color displays.

We also implemented large-scale tiling printing (6 × 4) exhibiting various colors (Fig. 7l). Color configuration was controlled by changing design parameters, i.e., photoresist thickness, metal type, and encoding conditions (Supplementary Table 2). For example, we examined the photoresist thickness in four conditions (100, 200, 300, and 400 nm) and observed that the color of the nanodisks red-shifted with increasing photoresist thickness. This behavior was most pronounced in Al nanodisks, which agrees well with our numerical simulations taking into account varying photoresist thickness (Supplementary Fig. 12). The thickness of photoresist can be controlled by experimental conditions (e.g., diluting ratio and spin-coating rate) and thus, be coated thinner than 100 nm, if necessary. Depending on the color, the thinner structure could appear more vivid. Al, Pt, Ag, and Au showed different color configurations: Au tended to shift the color to a more yellowish, Al to pink, and Pt and Ag to gray shades owing to their different plasma frequencies^51^. For encoding the original image to a plasmonic structure design, we constructed one pixel of an image with four subpixels consisting of nanodisk structures and adopted two modifications afterward. One was a nanodisk-free pixel to improve the white luminance thereby achieving the high contrast ratio (CR) and the other a subdivided nanodisk pattern to enrich the plasmonic color palette. This approach improved the image quality (Supplementary Fig. 13). All the plasmonic images with the same plasmonic structure design were patterned by the same photomask. We did not observe any deterioration in images or contamination on soft photomask which might be caused by repeated use of the photomask (Supplementary Fig. 14). PDMS is known to have poor adhesion with photoresist^52,53^ and thus, minimize contamination when the mask contacts to surface. Surface chemical treatment could further reduce contamination issue, if necessary^54,55^.

## Discussion

We demonstrate that our technique is a simple and powerful lithographic approach, which uses a photomask composed of chromium patterns embedded in elastomeric mask plate. Our soft photomask can be readily adopted in conventional photolithographic equipment and allows for the fabrication of feature sizes below the diffraction limit of light because of its near-field alignment with the surface. In combination with near-field ability for sub-wavelength feature generation, mechanically stable soft photomask provides high resolution and excellent reliability even on a highly curved surface. This advantage makes our technique a promising method for industrial production, especially for future non-planar electronics. This is a step towards the realization of a ‘routine nanofabrication technique’ that produces complex patterns on multiple length scales. The large-area fabrication of chromium patterns for soft photomask is inherently limited by the process of electron-beam lithography. However, considering the repeated use of mask, we infer that this parallel, high resolution, and low-cost patterning process will become an important alternative in the next-generation photolithography.

## Methods

### Fabrication of metal-embedded elastic photomasks

A silicon wafer (wet thermal oxide, 3000 Å, SEHYOUNG WAFERTECH) was cleaned with acetone and isopropyl alcohol. Ni thin film (200 nm) was deposited on the silicon wafer using a dc magnetron sputtering system. Electron-beam lithography was performed to define a mask pattern. After patterning, a Cr layer (30 nm) was sputtered onto the substrate, followed by a lift-off process using acetone. To improve the adhesion between the Cr layer and polydimethylsiloxane (PDMS, Dow corning, Sylgard 184), the substrate was treated with oxygen plasma at 100 W for 2 min. PDMS and a cross-linker were mixed (10:1 w/w) and degassed in vacuum. PDMS mixture was poured onto the substrate and cured at 80 °C for 4 h in an oven. As cured PDMS wrapped the whole substrate, PDMS was trimmed by a razor to expose the side of the Ni layer as sacrificial layer. It is then immersed into an aqueous iron chloride (FeCl_3_, reagent grade 97%, Sigma-Aldrich) solution at a concentration of 1 M for one week to completely etch the Ni layer. After cleaning with deionized water, the Cr-embedded PDMS slab was gently peeled off from the substrate and subsequently rinsed with deionized water.

### Fabrication of glucose sensor on a contact lens

The contact lens (NEON, Blue) was rinsed with DI water and dried by blowing nitrogen gas. As a supporting layer, SU-8 2025 (MicroChem) was coated and cured on the contact lens. A 100 nm-thick Au film with a 10 nm-thick Cr adhesive layer was deposited onto the contact lens. To define the electrodes, photoresist (Dow Electronic Materials, MEGAPOSIT SPR510A) is coated on the Cr/Au film and patterned with soft photomask. After development, Cr/Au film was etched by a gold etchant (Sigma-Aldrich) and a chrome etchant (Sigma-Aldrich). The patterned photoresist was removed by developer after exposing the UV light. CVD graphene (GRAPHENE SUPERMARKET) was transferred onto the electrode. SU-8 2002 (MicroChem) was coated onto the contact lens and patterned to confine sensing region. SU-8 layer was developed by rinsing with cyclopentanone (Alfa Aesar). As cyclopentanone can dissolve the contact lens, cyclopentanone should be rinsed within SU-8 supporting layer. Patterned sensing region was treated with methanol solution of 1-pyrenebutanoic acid succinimidyl ester by dropping the solution onto the contact lens, followed by cleaning with methanol. To immobilize GOD, the contact lens was immersed in GOD (10 mg/ml, Sigma-Aldrich, Glucose Oxidase from Aspergillus niger) and catalase (2.0 mg/ml, Sigma-Aldrich, Catalase from bovine liver) solution for 18 h, followed by cleaning with DI water.

To measure the resistance of the glucose sensor, one drop of glucose solution was transferred onto the glucose sensor for 30 s and gently blown by nitrogen gas to minimize current flow through excess glucose solution. We then connected the sensor to a parameter analyzer (Keithley, 4200A-SCS) by probe station (FormFactor, EPS-150FA) and the resistance of glucose sensor was measured by two probe method.

### Numerical simulations

Finite element analysis (FEA) was used to analyze the deformation of a metal-embedded soft substrate. All the mechanical analysis was carried out by the open-source FEA software Code_Aster. For the results shown in Fig. 1a, we adopted a 2D plane strain condition to simplify the simulation. The Young’s modulus (*E*) and Poisson’s ratio (*v*) were *E*_*Substrate*_ = 1 MPa and *v*_*Substrate*_ = 0.499 for the substrate and *E*_*Metal*_ = 100 GPa and *v*_*Metal*_ = 0.21 for the metal pattern. For Fig. 1b and Supplementary Fig. 17b, full 3D FEA was performed. The Young’s modulus (*E*) and Poisson’s ratio (*v*) were *E*_*PDMS*_ = 1 MPa and *v*_*PDMS*_ = 0.499 for PDMS and *E*_*Cr*_ = 140 GPa and *v*_*Cr*_ = 0.21 for Cr. Numerical simulation of the reflectance spectra of a nanodisk unit cell was carried out using COMSOL Multiphysics. In the 2D nanodisk array, the diameter of the nanodisk and the gap between adjacent nanodisks varied from 100 nm to 500 nm and from 150 nm to 450 nm, respectively. In the simulation, the thicknesses of Al, photoresist, and SiO_2_ were set to 20 nm, 200 nm, and 300 nm, respectively. The complex refractive indices of Al and Si were taken from ref. ^56^ and ref. ^57^, respectively. The refractive index of SiO_2_ was set to 1.46, and the refractive index of the photoresist was obtained by interpolation from three points. (1.744 for 365 nm, 1.689 for 436 nm, and 1.639 for 633 nm from the manufacturer of the photoresist).

## Supplementary information

Supplementary Information

## Data Availability

The data that support the findings of this study are available from the corresponding author upon request.
