# Supplementary material for: Near-field sub-diffraction photolithography with an elastomeric photomask
Source: Nat Commun. 2020 Feb 10;11:805. doi: 10.1038/s41467-020-14439-1 (PMC7010681; doi:10.1038/s41467-020-14439-1)
Supplement: Supplementary file 1 — Supplementary Information [file 41467_2020_14439_MOESM1_ESM.docx]

Supplementary Information

Near-field Sub-diffraction Photolithography with an Elastomeric Photomask

Paik et al.


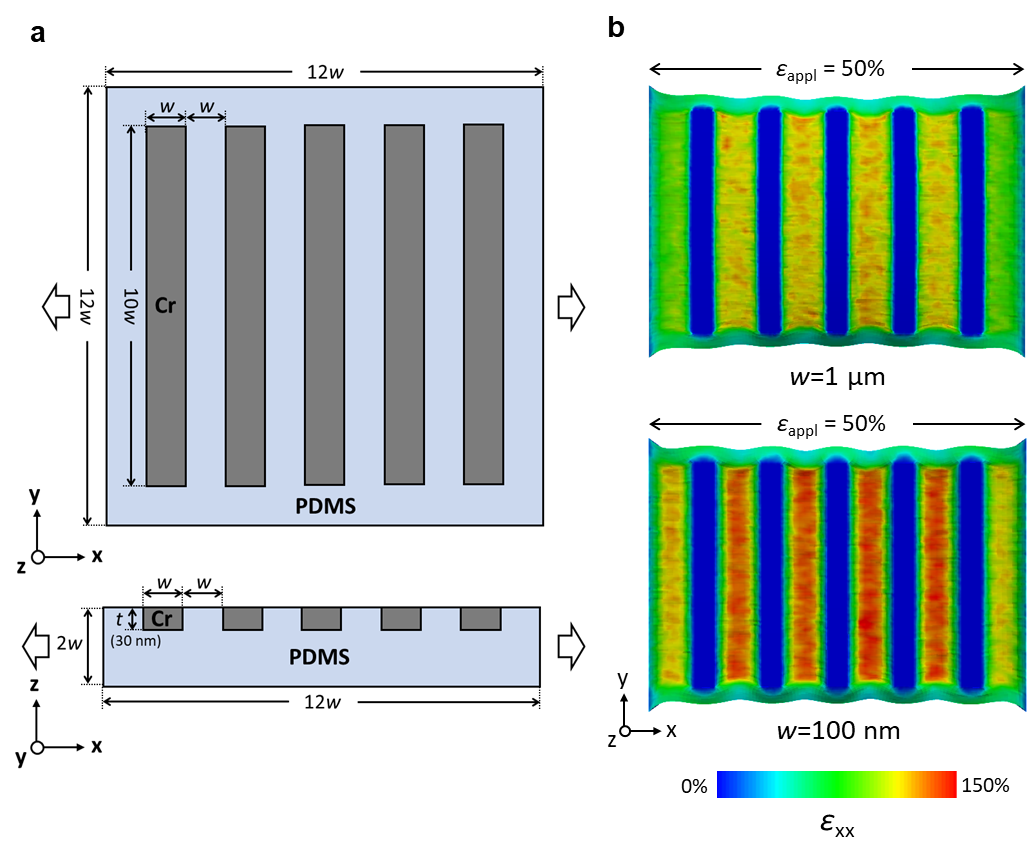


**Supplementary Figure 1 | 3D FEM simulation model. a,** Schematic illustrations of the 3D FEM simulation model. **b,** Simulated results of the strain (*ε*_xx_) distribution in a Cr line-embedded PDMS substrate.


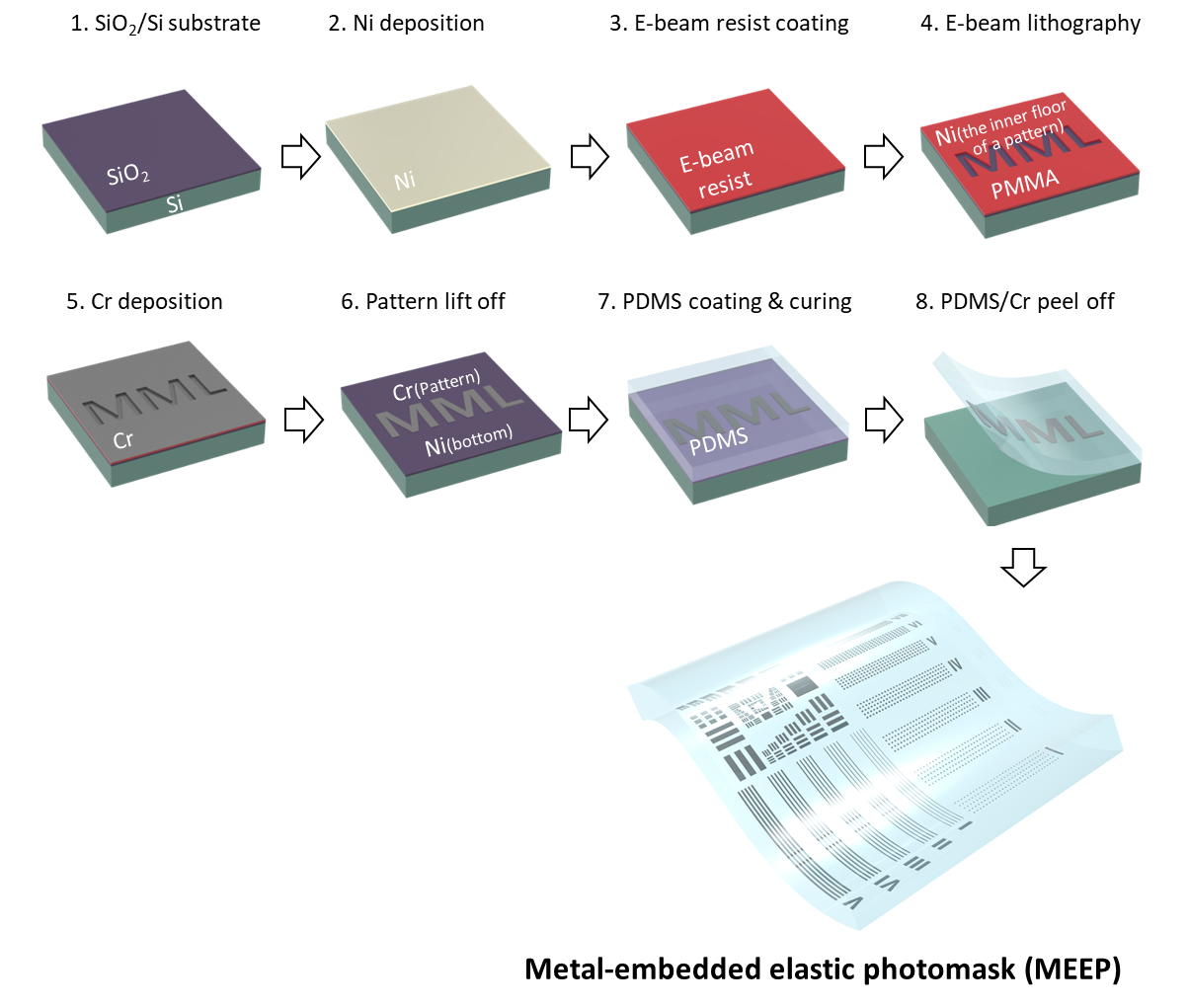


**Supplementary Figure 2 | Fabrication procedure of a metal-embedded elastic photomask.**


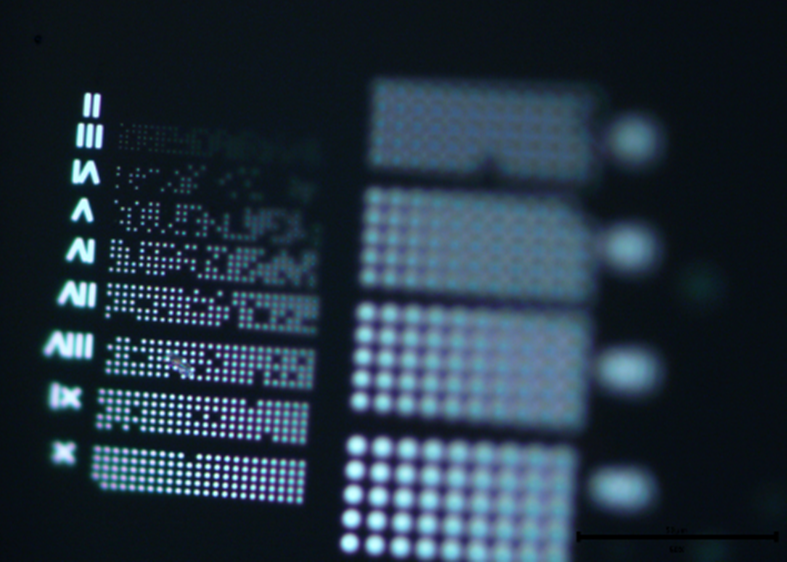


**Supplementary Figure 3 | Failed optical microscopy image of a soft mask because of debonding.**

**
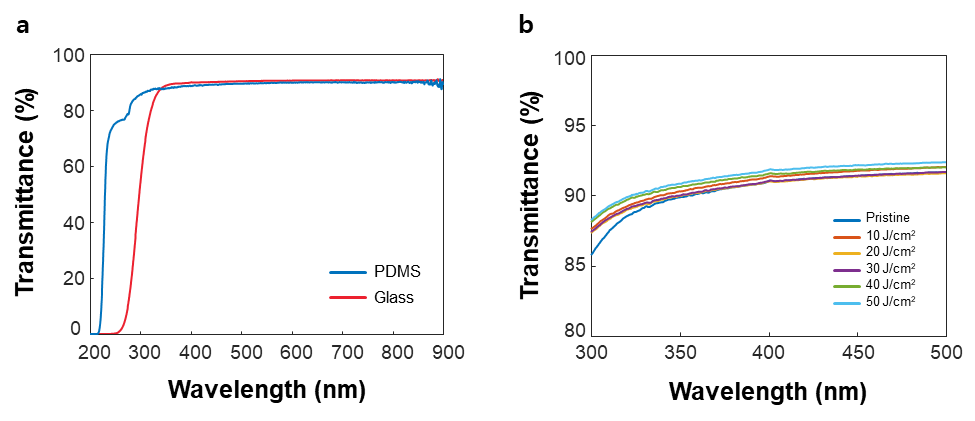
**

**Supplementary Figure 4** **| Optical properties of PDMS as a substrate of a soft photomask. a,** Optical transmittance of PDMS substrate and slide glass with a thickness of 1 mm. **b,** UV transmittance of PDMS substrate after being exposed to UV light with different doses.

**
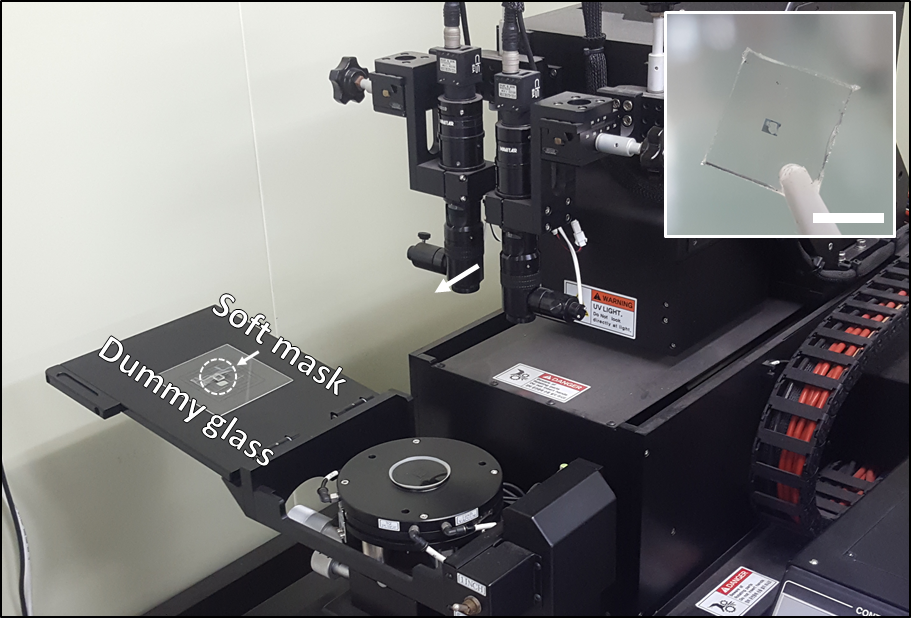
**

**Supplementary Figure 5 | Typical hardware setup to perform optical lithography process using soft photomask. Inset shows a picture of soft photomask. Scale bar in inset: 5 mm.**


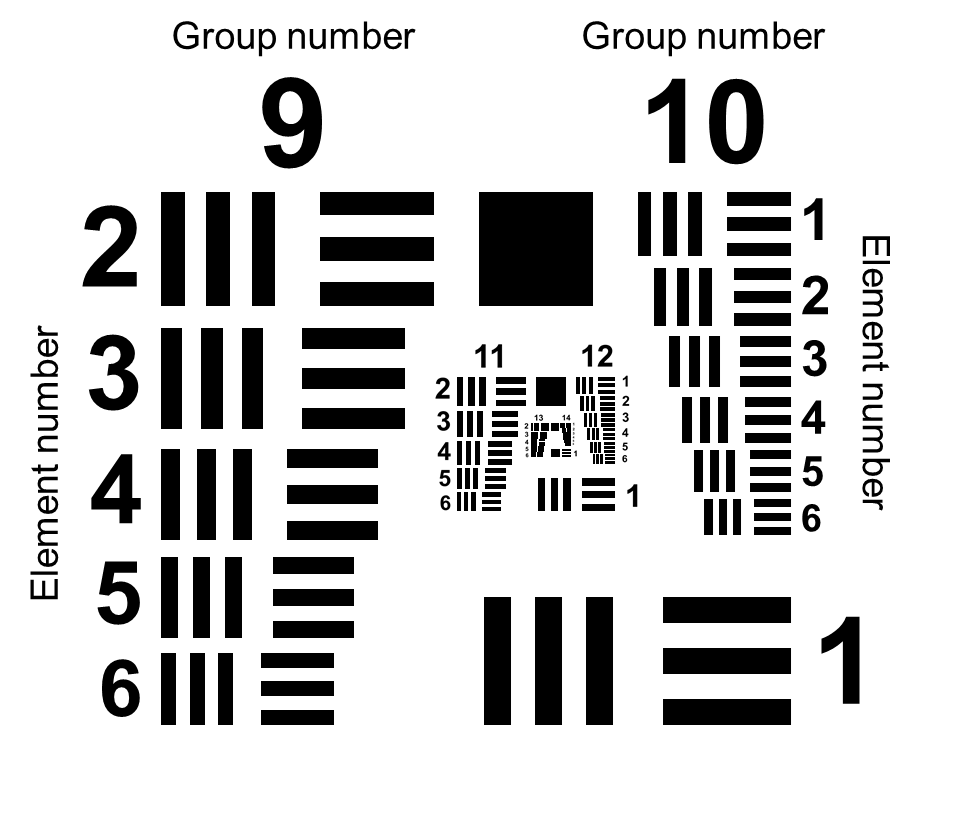


**Supplementary Figure 6 | Design of 1951 USAF (U.S. Air Force) resolution test chart for the resolution test.**

**
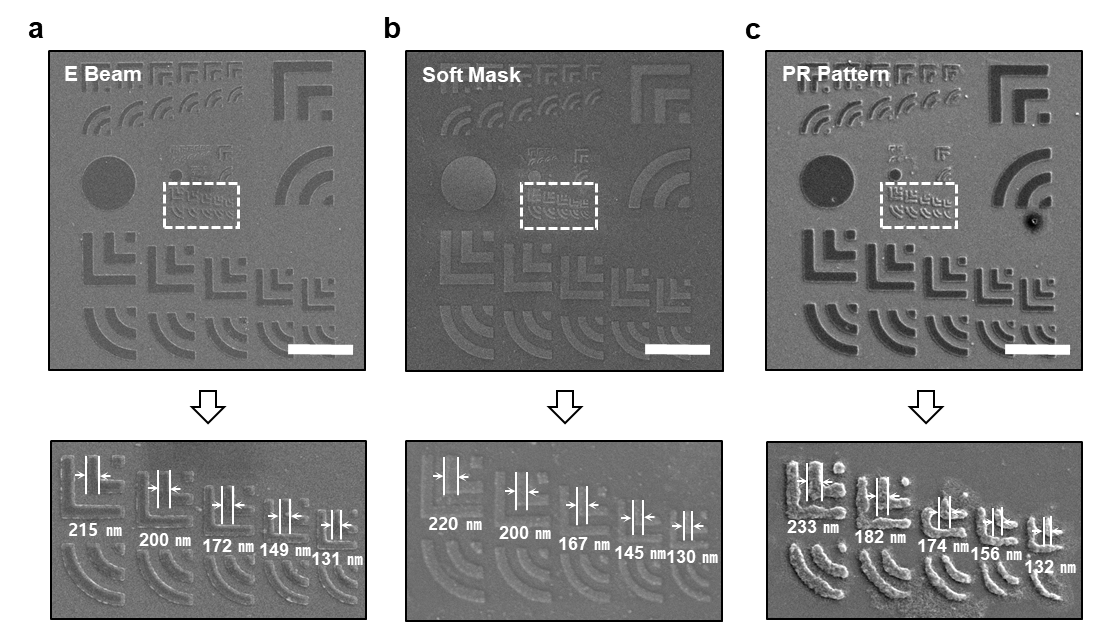
**

**Supplementary Figure 7** **| Modified 1951 USAF resolution test chart for arc and L-shaped pattern. a – c,** SEM images of the modified 1951 USAF resolution test chart at each transferring step illustrated in Fig. 4a, *i.e.,* defining Cr patterns by e-beam lithography (**a**), Cr patterns embedded in a PDMS mask (**b**) and the photoresist pattern (**c**). The lower panels show magnified images of the parts of the upper panel images indicated with dotted squares. Scale bars: 5 μm.

**
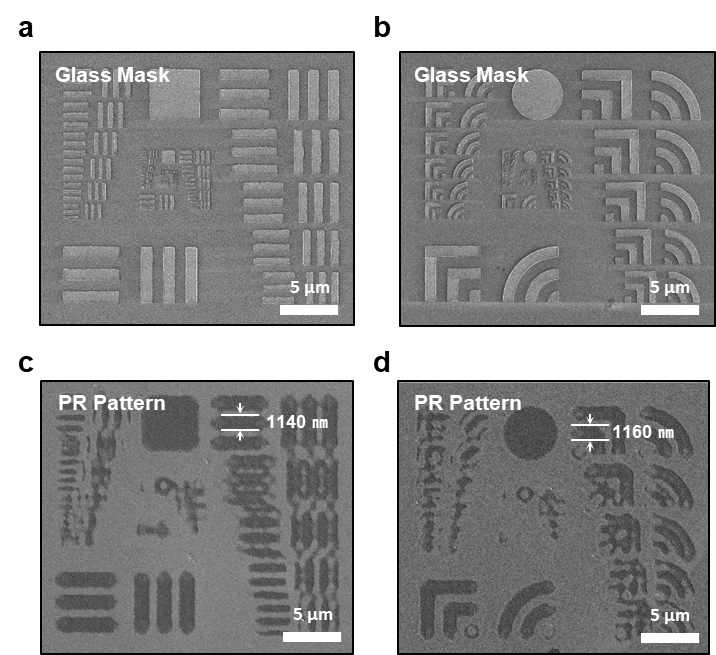
**

**Supplementary Figure 8** **| Resolution test with glass photomask. a, b,** SEM image of 1951 USAF resolution test chart pattern for the line (**a**) and arc and L-shaped pattern (**b**) in glass photomask. **c, d,** SEM image of photoresist features patterned by glass photomask with line (**c**) and arc and L-shaped pattern (**d**). Scale bars: 5 μm.

**
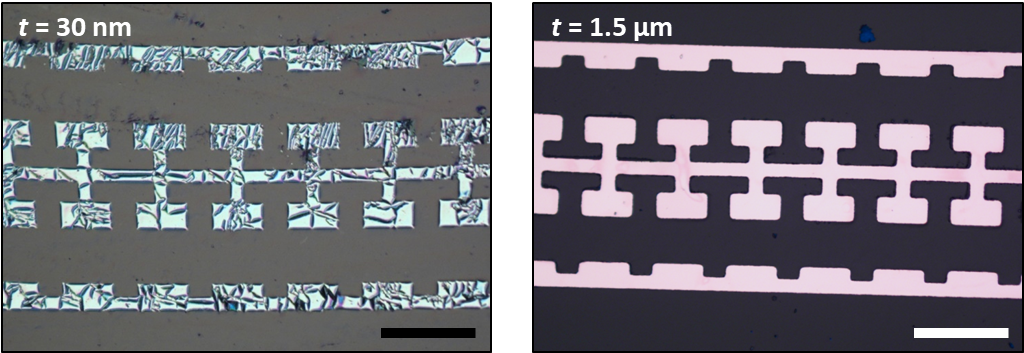
**

**Supplementary Figure 9 | Optical microscopy images of soft photomask with different thickness (*t*) of the metal pattern after being contacted with a photoresist-coated contact lens. Thicknesses of metal patterns were 30 nm (left) and 1.5 μm (right). Scale bars: 200 μm.**


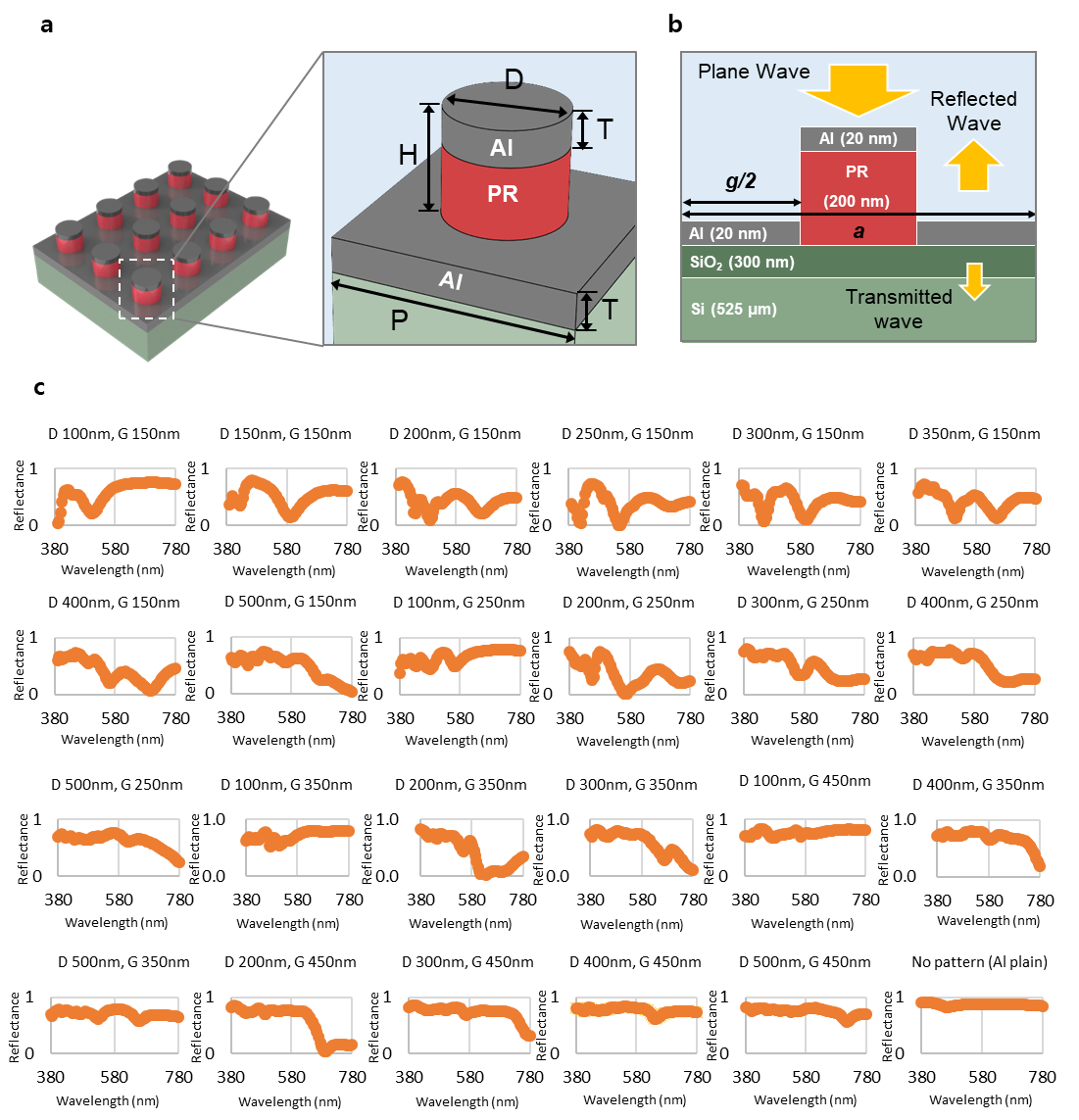


**Supplementary Figure 10 | FEM simulations of nanodisks of different structure. a,** Schematic illustration of a unit cell used in the FEM simulations. The nanodisks consist of a laminated structure of Si (525 μm)/SiO_2_ (300 nm)/Photoresist (PR, 200 nm)/Metal (20 nm), and the plane area consists of Si (525 μ m)/SiO_2_ (300 nm)/Metal (20 nm). **b,** Side view of the unit cell structure for simulation. We varied the disk diameter *D* and the gap between two adjacent disks *g*. **c,** Simulated reflectance spectra for each unit cell (*D*: 100-500 nm, *g*: 150-450 nm).


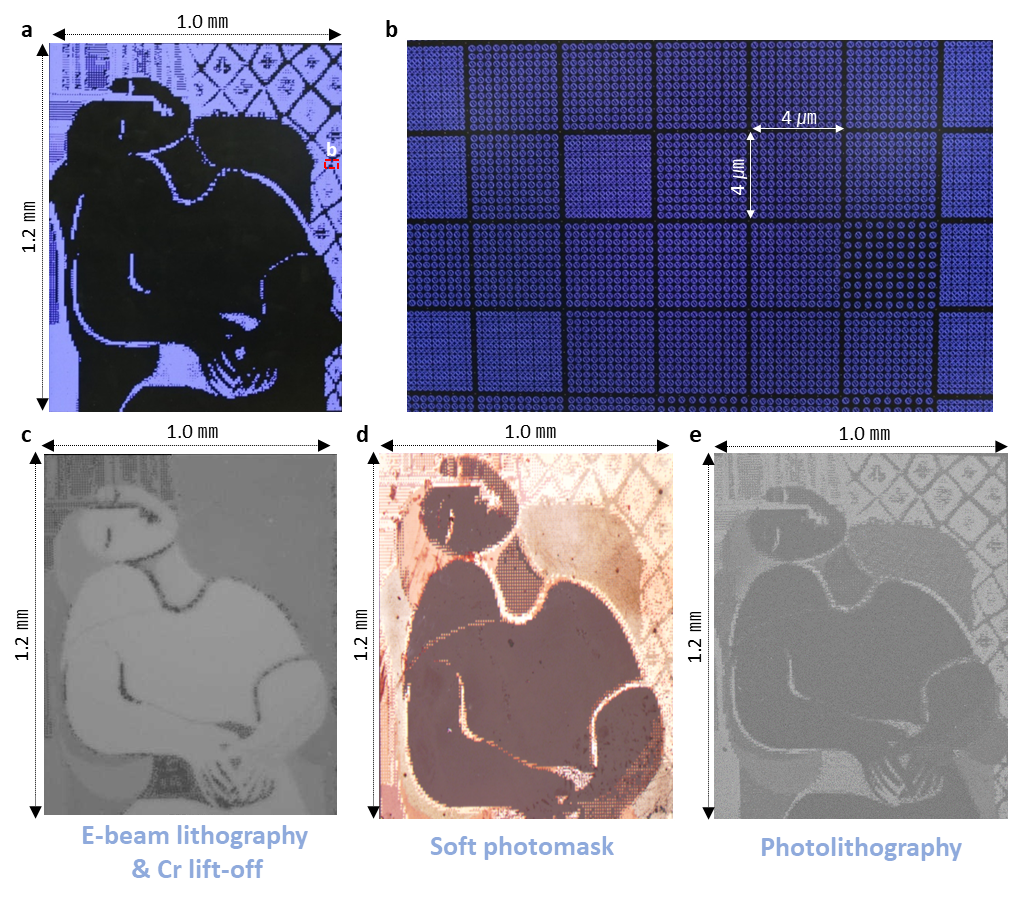


**Supplementary Figure 11 | Plasmonic structure design for color printing. a,** The design of the nanodisk arrays for a plasmonic color image consisting of 72,000 pixels. **b,** Magnified image of the plasmonic structure design. Each pixel consists of 16 to 255 nanodisks in each 4×4 μm^2^ area based on the color encoded data. **c,** SEM image of the Cr pattern formed through the lift-off process after electron-beam lithography on the Si substrate where Ni was deposited. **d,** Optical microscopy image of the Cr-embedded photomask. **e,** SEM image of the photoresist pattern after optical lithography using a soft photomask.


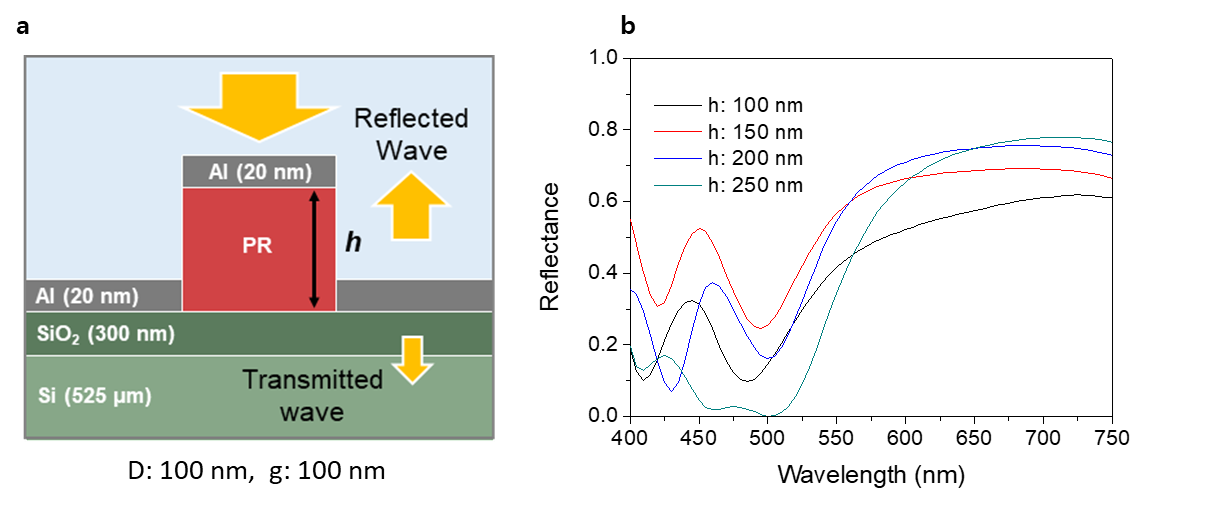


**Supplementary Figure 12 | Simulated reflectance depending on different photoresist thicknesses. a,** Schematic illustration of a unit cell used in the FEM simulations. **b,** Simulated reflectance spectra for different photoresist thickness.

**
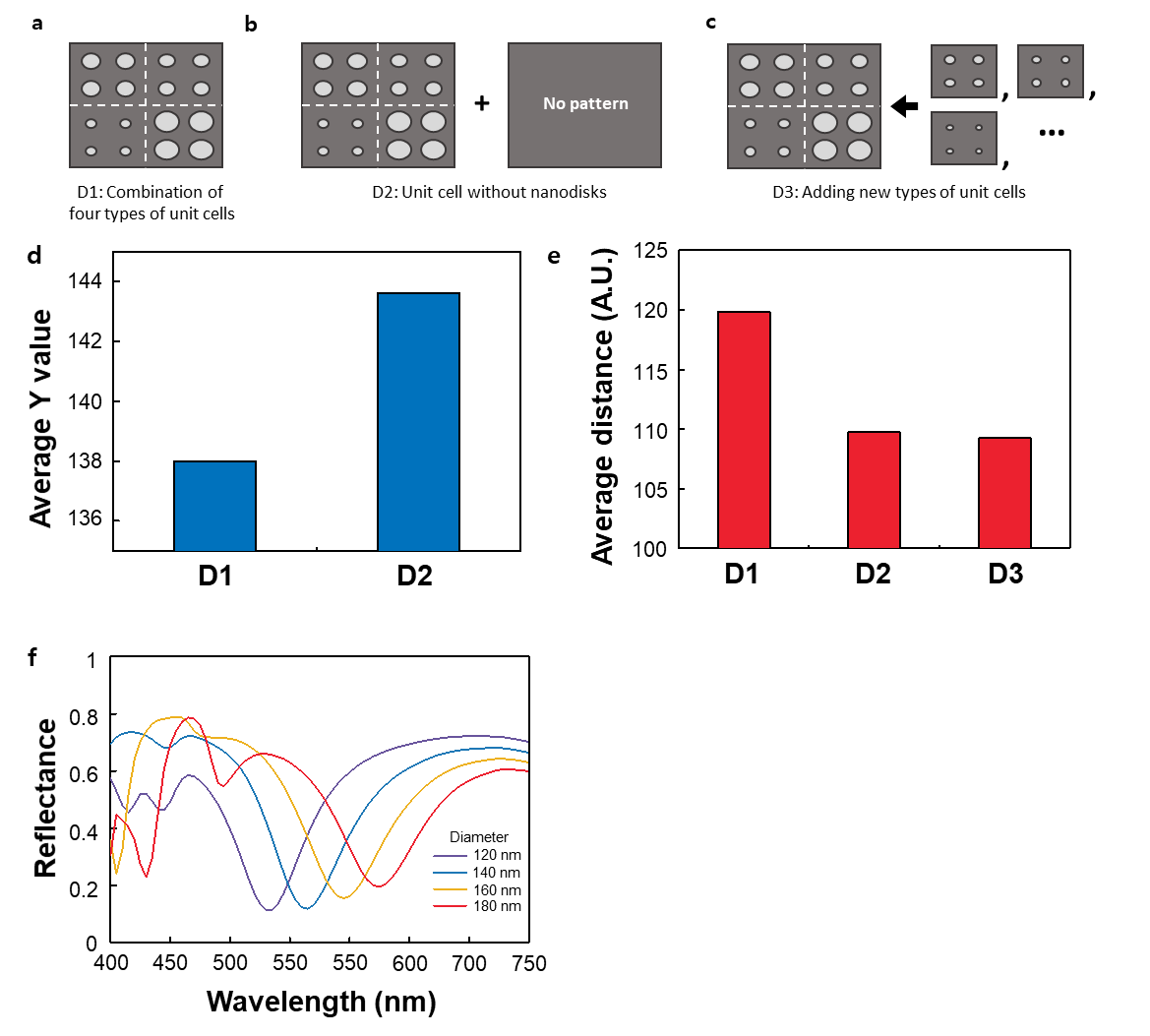
**

**Supplementary Figure 13 | Method for encoding an image to a plasmonic structure. a, b,** Schematic illustrations of designing methods for a plasmonic pixel. For encoding a whole image to a plasmonic structure, we prepared a color palette of plasmonic pixels, which consist of the unit cells investigated in Supplementary Figure 7. A plasmonic pixel was configured with four kinds of unit cells. We used 23 types of unit cell at first (D1, **a**), and then added a non-patterned pixel (D2, **b**) and additional types of unit cell (D3, **c**) to reduce the discrepancy between the original image and the plasmonic structure design. Each pixel of the original image was encoded in a plasmonic pixel whose reflectance spectrum best matched a pixel of the image based on the distance in RGB space. **d,** Average brightness (Y value) of the plasmonic structure design encoded by D1 (**a**) and D2 (**b**) in YUV space. **e,** Average distance of pixels between encoded designs and an original image in RGB space. **f,** Simulated reﬂectance spectra of additional nanodisk unit cells for D3 (**c**) with different diameters (*g*= 120 nm).


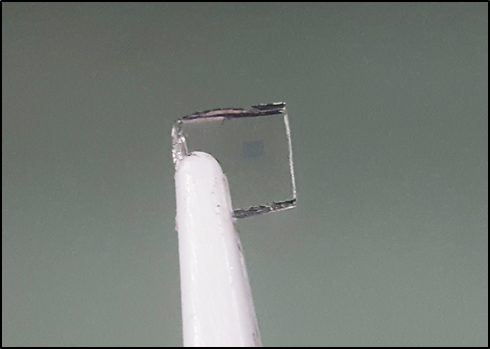


**Supplementary Figure 14** **| Soft photomask after patterning large-scale plasmonic nanodisks.**

**Supplementary Table 1 | Width of the lines in the 1951 USAF (U.S. Air Force) resolution test chart used for the resolution test**

| Element | Group Number | | | | | |
| --- | --- | --- | --- | --- | --- | --- |
| Number | 9 | 10 | 11 | 12 | 13 | 14 |
| 1 | 977 | 488 | 244 | 122 | 61 | 31 |
| 2 | 870 | 435 | 218 | 109 | 54 | 27 |
| 3 | 775 | 388 | 194 | 97 | 48 | 24 |
| 4 | 691 | 345 | 173 | 86 | 43 | 22 |
| 5 | 615 | 308 | 154 | 77 | 39 | 19 |
| 6 | 548 | 274 | 137 | 69 | 34 | 17 |

unit: nm

**Supplementary Table 2 | Process and design parameters of plasmonic images**

| Process and design parameter | | Number of plasmonic image |
| --- | --- | --- |
| Photoresist thickness | 100 nm | 1, 7, 13, 19 |
|  | 200 nm | 2, 3, 8, 9, 14, 15, 20, 21 |
|  | 300 nm | 4, 10, 16, 22 |
|  | 400 nm | 5, 6, 11, 12, 17, 18, 23, 24 |
| Metal element | Au | 1, 2 |
|  | Ag | 3, 4, 5, 6, 7, 8, 9, 10, 11, 12 |
|  | Pt | 13, 14, 15, 16, 17, 18 |
|  | Al | 19, 20, 21, 22, 23, 24 |
| Encoding algorithm | D1 | 12, 19, 22 |
|  | D2 | 2, 3, 4, 7, 8, 9, 10, 13, 15, 16, 20, 21, 23 |
|  | D3 | 1, 5, 6, 11, 14, 17, 18, 24 |

**Supplementary Text**

- The shear-lag model for a metal-embedded elastic photomask


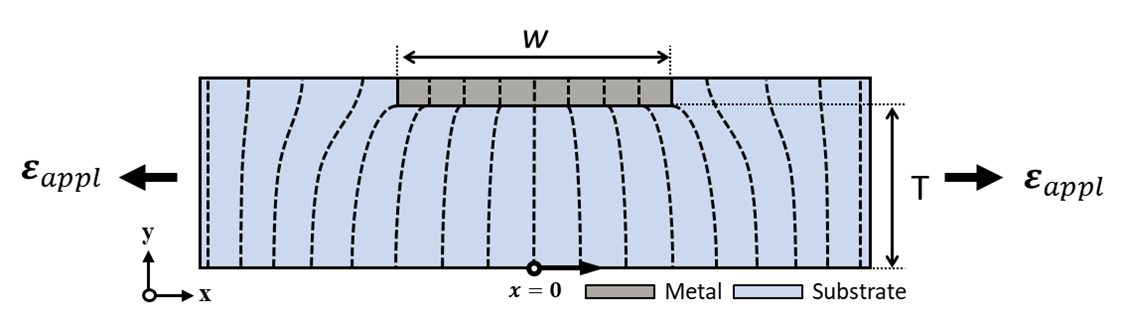


**Supplementary Figure 15 | Strain distribution in a metal-embedded soft substrate.**

In order to clarify the factors affecting a strain distribution of a soft mask, we derived an analytic equation adopting the shear-lag model. As we apply a strain to a metal-embedded soft substrate ($\varepsilon_{appl}$), the substrate shows different deformation behaviors depending on the distance to the metal thin film because of the different Young’s moduli in metal and substrate (Supplementary Figure 15). This distorted deformation profile induces the shear stress. In this model, we assumed that the strain applied to the substrate is transferred to the metal thin film by the shear stress at the metal/substrate interface.

The effect of the metal thin film to deform the substrate becomes smaller as the distance from the interface increases. We defined the effective thickness *T* of the substrate as behind the effective thickness *T*, the distortion of deformation caused by the metal thin film disappears therefore we don’t need to consider a shear stress. Assuming that the shear stress in the substrate is uniform along the y-axis to simplify the model, the shear stress at the metal/substrate interface can be described as

$\tau_{i}=G_{s}\frac{u(x)-u_{T}(x)}{T}$, (S1)

($\tau_{i}$: shear stress at the interface, $G_{s}$: shear modulus of the substrate, $u$: displacement)


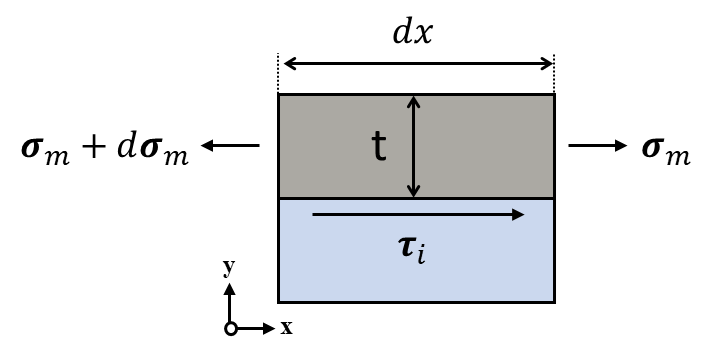


**Supplementary Figure 16 | Force balance at the metal/substrate interface.**

Where,

$u_{T}(x)=\varepsilon_{appl}x$. (S2)

The shear stress at the interface is balanced with the variation of tensile stress in the metal thin film along the x-axis (Supplementary Figure 16). A force balance can be described as

$\tau_{i}dx=td\sigma_{m}$. (S3)

($\sigma_{m}$: tensile stress in the metal, $t$: thickness of the metal)

Hence,

$\tau_{i}=t\frac{d\sigma_{m}}{dx}=E_{m}t\frac{d^{2}u}{dx^{2}}$. (S4)

From (S1) and (S4), a second order ordinary differential equation can be derived as

$\frac{E_{m}Tt}{G_{s}}\frac{d^{2}u}{dx^{2}}=u(x)-\varepsilon_{appl}x$. (S5)

by two boundary conditions,

$u(0)=0$, (S6)

and

$\sigma_{m}(\frac{w}{2})=E_{m}{\frac{du}{dx}}_{x=\frac{w}{2}}=0$. (S7)

Equation (S6) can be solved by


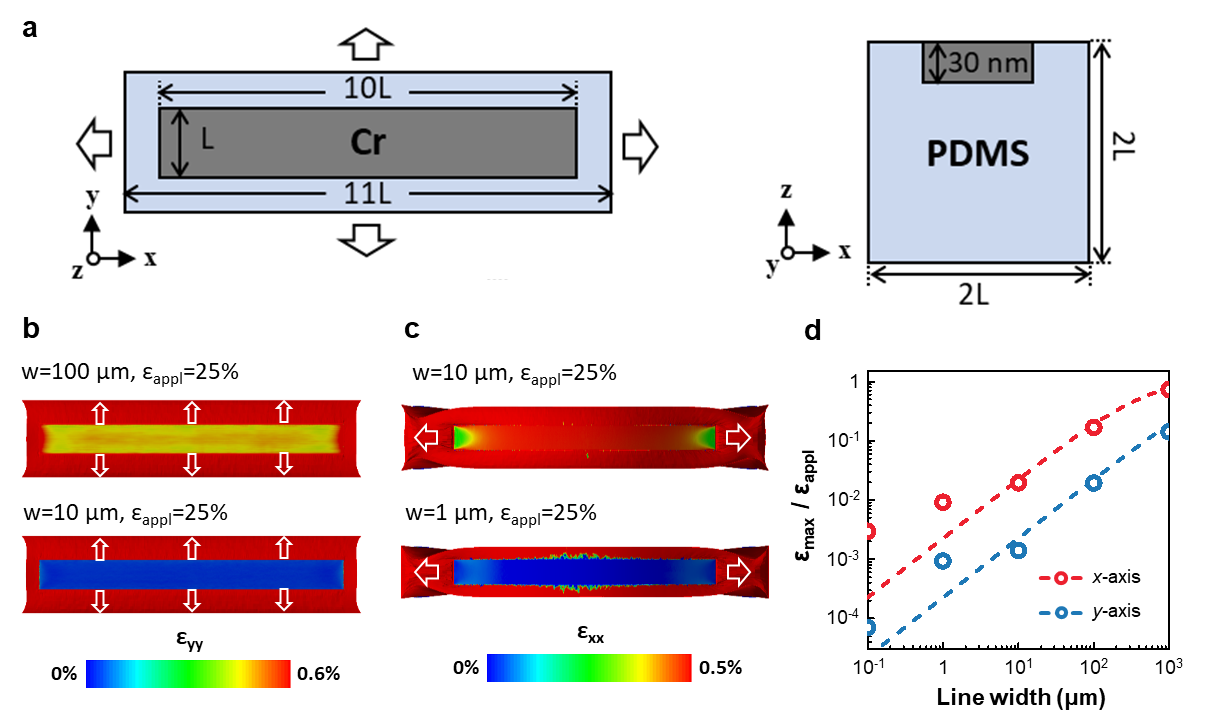


**Supplementary Figure 17 | Applying the model to a 3D FEM simulation result. a,** Schematic illustrations of the 3D FEM simulation model. The Young’s modulus (*E*) and Poisson’s ratio (*v*) were *E_PDMS_*=1 MPa and *v_PDMS_*=0.499 for PDMS and *E_Cr_*=140 GPa and *v_Cr_*=0.21 for Cr. **b, c,** Simulation results of the strain distribution in the Cr line-embedded PDMS substrate. The substrate was stretched along the y-axis (**b**) and x-axis (**c**). **d,** The ratio of the maximum tensile strain in the Cr line to the strain applied to PDMS substrate depending on the line width of the Cr line. The circles denote the 3D FEM simulation results for the x-axis (red) and the y-axis (blue) and the dashed line was calculated using the shear lag model.

$u(x)=\varepsilon_{appl}\left[ x-\alpha\frac{\sinh\left( \frac{x}{\alpha} \right)}{\cosh\left( \frac{w}{2\alpha} \right)} \right]$, (S8)

where,

$\alpha=\sqrt{\frac{E_{m}Tt}{G_{s}}}$. (S9)

Differentiating (S8) with respect to x, the strain in the metal $\varepsilon_{m}(x)$ can be derived as

$\varepsilon_{m}(x)=\frac{du}{dx}=\varepsilon_{appl}\left[ 1-\frac{\cosh\left( \frac{x}{\alpha} \right)}{\cosh\left( \frac{w}{2\alpha} \right)} \right]$. (S10)

The maximum tensile strain of the metal thin file is built at $x=0$,

$\varepsilon_{max}=\varepsilon_{appl}\left[ 1-\frac{1}{\cosh\left( \frac{w}{2\alpha} \right)} \right]$. (S11)

Therefore,

$\frac{\varepsilon_{max}}{\varepsilon_{appl}}=1-\frac{1}{\cosh\left( \frac{w}{2\alpha} \right)}=1-\frac{1}{\cosh\left( \sqrt{\frac{G_{s}w^{2}}{4E_{m}Tt}} \right)}$. (S12)

To validate the model, we applied it to 3D FEM simulation results (Supplementary Figure 17). In this simulation, we stretched Cr line-embedded PDMS substrates along the *x*- and *y*-axes with different Cr-line widths and derived the ratio of the maximum tensile strain in a Cr line to the strain applied to the PDMS substrate. Since the effective thickness *T* is not determined by explicit factors such as geometry and material properties, we should redefine the effective thickness before applying equation (S12) to this case. We simply assume that the effective thickness *T* is proportional to the width of the metal film ($T=w/a$). Therefore,

$\frac{\varepsilon_{max}}{\varepsilon_{appl}}=1-\frac{1}{\cosh\left( \sqrt{\frac{{G_{s}w}^{2}}{4E_{m}Tt}} \right)}=1-\frac{1}{\cosh\left( \sqrt{\frac{aG_{s}w}{{4E}_{m}t}} \right)}$. (S13)

We found the constant *a* by fitting equation (S13) to 3D FEM simulation results (Supplementary Figure 17d). In this case, we achieved the best curve fit with $a=16.28$. For the strain applied along both *x*- and *y*-axis, the calculated results using the shear lag model were in good agreement with the 3D FEM simulation results. This suggests that the deformation behavior of soft mask is well described by the shear-lag model we derived.
